# On the Rights of Physicians and Apothecaries

**Published:** 1813-04

**Authors:** 


*To the Editors of the Medical and Physical Journal.*

GENTLEMEN,

THE impartiality that you not only profess but exercise, joined to your own talents and industry, have filled your pages with valuable information, and deservedly placed you among the highest of the medical journalists. Permit me then, through your medium, though perhaps differing in sentiments from yourselves, to offer to my brethren a few observations relative to the important question now agitated by the Apothecaries.

It

It is well observed by Censorinus, and it is a fact of which every medical man must be aware, that "the province of attending on, and prescribing for, the sick, has been usurped and pertinaciously maintained by the apothecaries."[1] Hence it follows, that the regular physician is not in fault, since he still sends his prescriptions to the legitimate pharmacopolist; but it is the apothecary who has "overstepped the modesty" of his part of the profession; and, having departed from the sphere in which he ought to have acted, has certainly no right to complain. The increasing influence of the apothecaries has always struck me as pregnant with serious evil. Are not these stern assertors of their own rights already satisfied with the wrongs they have inflicted on the profession? But now, that they aim at nothing less than obtaining the whole circle of practice, that physician denies his duty who does not, by his voice, his influence, or his pen, endeavor to destroy their league. How many an amiable and highly-cultivated mind is left to languish in obscurity and misery, while the ignorant apothecary lolls in his chariot, prescribes to his own shop, and laughs at the peripatetic graduate!

It is a well-known fact, that an apothecary seldom calls in a physician but under one of three cases: either when he knows not what more to do, when the friends insist, or when the patient will take no more medicine. I have heard several of their body declare, that they never would call in a physician. Many a valuable life is thus lost to the community. The physician comes but to be followed by the undertaker—he comes but to confirm the apothecary's declaration, "that nothing more can be done!" While, had the man of corks and vials honestly played his part, the tears of many an orphan, the sorrows of many a friend, would have been spared. Who foresees not the consequences of their present intentions? by establishing a legal claim for fees, they obtain an advantage over the regular practitioner; their charges, at first moderate, will gradually increase, till few can afford the expense of physician and apothecary. From the very nature of their part of the profession, they will be *generally* the first employed. "Oh, but (says the apothecary) there is less need than ever for a physician—I am now a well-informed man—look at my license!" As their system of education is cheaper, and the advantages more immediate, their numbers will be constantly increasing, till the physicians are absolutely overwhelmed; and then, instead of health restored, I confidently predict that another box of Pandora will be opened upon the world. I will not now encroach so far upon your valuable pages as to enter upon a complete examination of their plan; if this letter is received, that may serve serve for the occupation of a future hour; far be it from me to oppose any improvement in their education—let them only be confined to their proper sphere—let them compound, dispense, and deliver. "*Hæ tibi erunt artes*"—but beware of prescribing. Let us all retain our proper places, and the medical profession will afford fame for many, and a fair reward for all: but, when the floodgates are removed, and the rushing torrent breaks down its banks, I confess I see nothing but desolation and ruin for the country round.

I am, Gentlemen,

Your humble Servant,

SALUS PUBLICA.

---

*To the Editors of the Medical and Physical Journal.*

